# Effect of low-level laser therapy in wound healing of primary molar teeth extraction

**DOI:** 10.1186/s12903-024-04145-5

**Published:** 2024-03-19

**Authors:** Hazal Özer, Merve Abaklı İnci

**Affiliations:** Department of Pediatric Dentistry, Faculty of Dentistry, Necmettin Erbakan University, Yaka Mahallesi Bağlarbaşı Sokak, 42090 Meram, Konya Turkey

**Keywords:** Low-level laser therapy, Pain assesment, Pediatric dentistry, Tooth extraction, Wound healing

## Abstract

**Introduction:**

Tooth extraction in children requires attention to wound healing and pain management, which are influenced by patient-related factors and behavioral guidance. Aim of the study: The study aimed to evaluate the effect of LLLT on healing sockets in pediatric patients with bilateral primary molar teeth extraction and determine its impact on pain management.

**Methods:**

6–10 years of age, systemically healthy, and with atraumatic extraction indications of bilateral primary molar teeth were included in the study (*n* = 40). In the first session, randomly selected teeth were extracted under local anesthesia. In the control group, only clot formation in the socket was observed and photographed. The other group extractions were performed 2 weeks later. The low-level laser therapy (LLLT) group was treated with a 980 nm wavelength, in a continuous emission mode, 0.5 W power, 300 J of energy, 400 µm tip, 60 s diode laser and photographed. Nonepithelialized surface measurements were performed using ImageJ. Pain assessment was performed using the Wong-Baker Pain Scale. Statistical analyses were performed using SPSS software.

**Results:**

There was a statistically significant difference between the groups in the Wong-Baker values in 3rd day (*p* < 0.05). In soft tissue healing on the 3rd and 7th day, the nonepithelialized surface of the laser socket was smaller than that of the control group, and the measurement results were found to be statistically significant (*p* < 0.05).

**Conclusion:**

Although LLLT was not found to be very effective in reducing postoperative discomfort after extraction of primary molars, it provided better wound healing in extraction sockets.

## Introduction

Tooth extraction may be an unpleasant and painful experience for children [1]. Pain felt during dental treatment, especially tooth extraction, is the most common complication. Dental fear and anxiety occur in pediatric patients whose pain control cannot be achieved during or after tooth extraction [2]. Postextraction wound healing and pain perception are related to host-related factors, such as the patient's immune system, the use of painkillers and antibiotics, the presence of infection in the region, and the atraumatic nature of the procedure. Trauma to the area whose numbness continues after local anesthesia by biting can also cause inflammation and pain in that area after extraction. Pain management during and after tooth extraction is part of behavioral guidance, especially in pediatric patients [3, 4].

Low-level laser therapy (LLLT) is a widely used adjuvant treatment for wound healing [5]. It is based on the idea that exposure to a specific wavelength can alter cellular behavior, resulting in both an increase in cell number and an increase in cell metabolism [6]. Aside from not having any negative effects, LLLT is an athermic, photobiological, and nondestructive therapy approach. Low-level working lasers typically operate at wavelengths of 630–980 nm and in the 50–550 mW range. They are also smaller and more cost effective. These therapeutic lasers are used in a process known as "low-level laser therapy," "biostimulation," or "biomodulation." Herpes simplex, mucositis, postsurgical pain and inflammation prevention delay, and dysfunction of the temporomandibular joint are conditions in which therapeutic lasers are used [7, 8].

The widely acknowledged rationale behind improving tissue repair is that laser devices provide energy target cells can utilize to activate their membranes or organelles. Cytochromes in the mitochondria absorb laser radiation, converting it into energy through cell adenosine-5-triphosphate. This, in turn, plays a role in protein synthesis and accelerates or stimulates cell proliferation. At the cellular level, increased cell proliferation and enhanced mobility of fibroblasts and keratinocytes are commonly observed after laser irradiation, holding notable significance for wound healing [9, 10].

Another consequence of LLLT reported in vivo is that it boosts macrophage phagocytic activity during the early phases of tissue formation after damage. This indicates that wound debridement was facilitated, creating circumstances for the proliferative phase of healing to begin [11]. Histologically, a blood clot forms and transforms into granulation tissue in the first 6–8 weeks after extraction, which is eventually replaced by mineralized and immature bone. During this time, applying LLLT to the extraction socket has no side effects; instead, it stimulates an inflammatory response that promotes tissue repair. It is defined as a biostimulation method that accelerates wound healing and thereby eliminates postextraction discomfort [12].

It is crucial to emphasize that LLLT also enhances the wound-healing process in immunosuppressed individuals. This presents a significant treatment option for patients with compromised healing capacities, including those with diabetes, HIV, or those undergoing radiotherapy. An animal study involving rats subjected to radiotherapy indicated that the application of 830 nm GaAlAs diode laser at 75 mW immediately after tooth extractions expedited bone healing. At the same time, the control group experienced a delay in the bone healing process. This research demonstrated the histological presence of mature collagen fibers, early formation of new bone, and histomorphometric analysis revealing an increase in the area of bone trabeculae in the alveolus following laser irradiation [13].

Furthermore, a clinical study involving individuals who are HIV-positive revealed an expedited post-extraction neoangiogenesis following daily application of diode laser (820 nm) for a duration of 5 days post-tooth extraction. This accelerated neoangiogenesis is significant for promoting the wound healing process. Additional clinical trials that assess various forms of LLLT in immunosuppressed patients are necessary to provide evidence supporting its reliability as an adjunct therapy following tooth extraction [14].

The purpose of this study was to clinically evaluate the effect of LLLT on healing sockets in pediatric patients who are scheduled to have bilateral primary molar teeth extracted, as well as to determine whether it influences pain management. The study confirmed that low-level laser therapy (LLLT) promotes faster healing in pediatric extraction sockets compared to the control group, and less pain was reported at the extraction site.

## Methods

### Study design

This double-blind, split-mouth, randomized clinical trial was designed to evaluate the effectiveness of LLLT on the postoperative results of primary molar tooth extractions in patients who were treated at the Dentistry Department of Pedodontics between November 2017 and April 2018. Our study was approved by the Ethics Committee of Necmettin Erbakan University Faculty of Dentistry, numbered 2017–07.

The minimum number of teeth to be evaluated in the study was 35 according to the G Power test (G*Power Software, Ver.3.1.) applied considering the data obtained in the sample studies, but *n* = 40 was used to avoid loss of patient-related data (*α* = 0.05, *β* = 0.20).

This research was registered with Clinical Trials ID NCT06018584 (30/08/2023).

Our study included 80 children aged 6–10 years who did not have any systemic disease and had a Frankl behavioral scale score of 3 or 4 [15]. The procedures and their adverse effects were thoroughly described to the parents of the pediatric patients, and an informed consent form was reviewed and signed by the parents, who also received a copy.

### Inclusion and exclusion criteria for patients and teeth

As a result of clinical and radiographic evaluations of pediatric patients, it was considered that there was an indication for tooth extraction in bilateral primary molars, that they did not have any systemic disease and that the use of antibiotics and painkillers was stopped at least 12 h prior. During radiographic evaluation, attention was given to the fact that bilateral primary molars do not require complicated tooth extractions, do not show signs of infection and have almost the same level of indications for atraumatic extraction. The research focused on teeth that had undergone at least two-thirds of the root formation process.

The study excluded children who either had no telephone contact number provided by their parents for supervision during the postoperative period or had a medical history involving conditions such as prolonged bleeding, platelet disorders, hypersensitivity, allergic reactions to pain relievers, contraindications to laser therapy, and acute pain.

### Determination of working groups

**Fig. 1:**
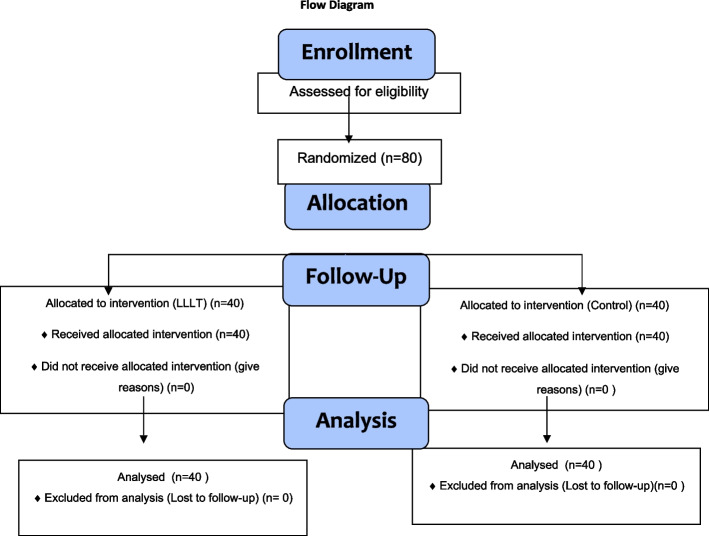
Participant flow chart

Teeth were randomly assigned to either the control or LLLT group before the allocation (Fig. 1). The group of teeth that were extracted in the first session was determined randomly using R 2.11.1 software (R Foundation for Statistical Computing, Vienna, Austria). Randomization was performed by a nonpractising physician. The practitioner was not blinded to the groups. By performing the same treatments on both teeth without turning on the laser in the control tooth, the patients and their parents were rendered blind to the groups.

### Local anesthesia and tooth extraction

The tooth extractions were performed by a single physician. 10% lidocaine spray as topical anesthesia was applied to the dried mucosa in the area to be localized with the help of an ear stick for 1 min. Local anesthesia was attained through the administration of 2% articaine hydrochloride combined with 1/100,000 epinephrine. Posterior-superior-alveolar nerve block anesthesia and palatal local infiltration anesthesia were applied to the upper primary molars, and inferior alveolar nerve block anesthesia and lingual nerve anesthesia were applied to the lower primary molars with an average injection dose of 1.5–2 ml. After control of anesthesia was achieved, a randomly selected tooth was extracted. Two weeks later, tooth extraction on the other side was performed. During shooting, attention was given to the atraumatic approach. After controlling for bleeding, data were recorded for each group.

### Approach after tooth extraction

In the LLLT group, the diode laser device Doctor Smile Wiser (Wiser, Doctor Smile, Milan, Italy) with a wavelength of 980 nm and a power of 0.5 W was used for LLLT. During the procedure, the patient, physician, and assistant staff wore protective glasses (Wiser Doctor Smile, Milan, Italy). With 300 J of energy, in a continuous emission mode, a 400 µm tip held 1 cm away from the extraction socket was solely applied to the extraction socket for 60 s from three points determined from the vestibule, lingual/palatal, and occlusal surfaces.

**Fig. 2:**
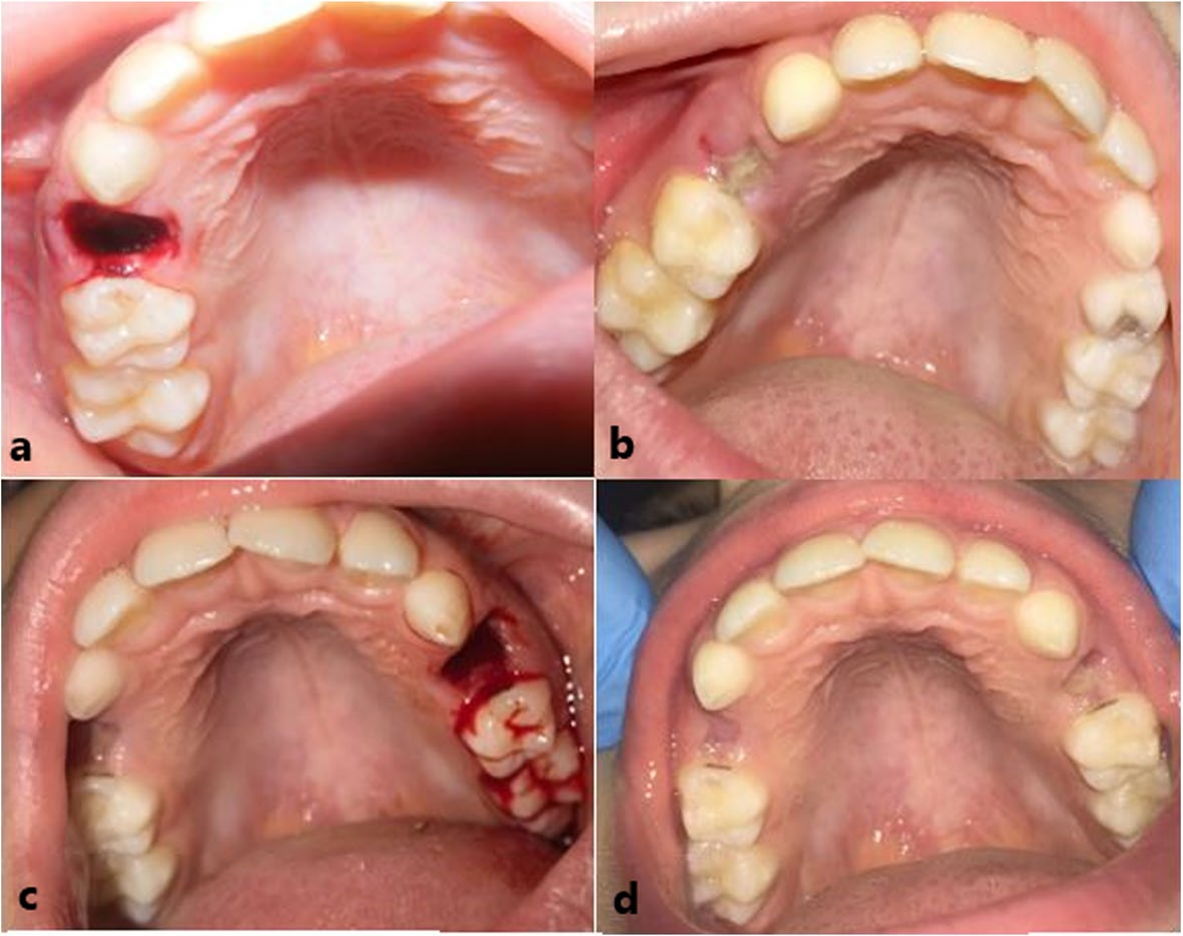
**a** Extraction socket at the 1st day of LLLT group. **b** Extraction socket at the 3rd day of LLT group. **c** Extraction socket at the 1st day of control group. **d** Extraction socket at the 7th day of control group

After tooth extractions in the sham group, no actual laser application was performed. However, the sound produced by the laser device during operation was prerecorded on a mobile phone. As a control group procedure, the application was mimicked by playing the recorded sound in the area without activating the device. This was done to create the impression for the patient who the procedure was applied to both tooth sockets (Fig. 2).

All pediatric patients and their parents were provided information about postextraction care, and appointments for control sessions were scheduled. The danger of damaging the soft tissue of the child's teeth, which is one of the complications of local anesthesia, was emphasized again, and the parent was instructed to notify us if this occurred.

### The wong-baker faces pain rating scale assessments in study groups

**Fig. 3:**
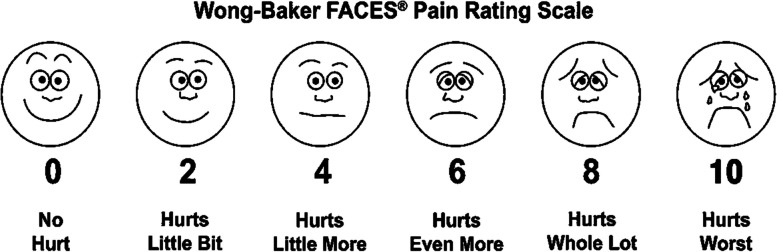
Wong-Baker FACES® Pain Rating Scale. Wong-Baker FACES Foundation with permission from http://www.WongBakerFACES.org

Pain assessment in both groups was performed with the Wong-Baker Pain Rating Scale (Wong-Baker FACES Pain Rating Scale PRS) (Fig. 3). Patients were instructed to indicate their pain level by selecting a face or number from the scale, and the chosen value represented the perceived pain 3 h after the dental procedure on that particular day; for all other days, the parents marked the pain scores on behalf of their children. During the seven days following the extraction session, the parents informed the doctors about the values chosen by the child patients from the scale given to them, the need for painkiller use, and the number of uses. Pain formation as a result of trauma to the soft tissue by biting, which is a complication of local anesthesia, was not recorded.

### Wound healing assessment

To evaluate soft tissue healing in extraction sockets, intraoral photographs were taken at an angle of 60° to the occlusal surface on the 3rd and 7th days following the extraction session. Nonepithelialized surface measurements were performed after appropriate calibrations by three observing physicians using ImageJ software (NIH, Rockville, USA).

### Statistical assessment

Statistical evaluations of the obtained data were performed using SPSS software (SPSS 21.00; IBM, Chicago, USA). The data were analyzed using the χ^2^ (chi-squared) test and Wilcoxon signed-rank test for categorical data analysis. Pairwise comparisons between parental ratings and children's ratings were conducted using Spearman's rank correlation coefficient. A significance level of *p* < 0.05 was set for statistical significance.

## Results

The study was conducted with 160 teeth in 80 children [45 girls (56.25%), 35 boys (43.75%)] 6–10 years of age (mean: 9.11–1.089). 94 of 160 teeth were maxillary primary molars (58.75%), and 66 were mandibular primary molars (41.25%). Low-level laser therapy had a statistically significant effect on accelerating wound healing compared to the control group on the 3rd and 7th days (Table 1). Additionally, the Wong-Baker pain scale values showed that pain reported on the 3rd day was also statistically significant when compared to the control group (Table 2).

**Table 1:** Non-epithelialized surface diameters on the extraction day, 3rd and 7th days

Surface Diameters (mm^2^)	LLLT Group (*n* = 40)	Control Group (*n* = 40)	*p*
T0	6.82 ± 1.52	6.34 ± 1.77	0.31
T1	3.82 ± 2.02	4.79 ± 1.60	0.0017*
T2	2.40 ± 0.5	3.2 ± 0.5	0.0001*

T0:1st day T1: 3rd day T2:7th day

^*^p < 0.05

**Table 2:** Wong-baker pain rating scale data on the extraction day, 3rd and 7th days

WB-Pain Rating Scale Medians	LLLT Group (*n* = 40)	Control Group (*n* = 40)	*p*
T0	4 ± 1.9	4 ± 2.07	1
T1	1 ± 0.72	3 ± 1.38	0.0001*
T2	1 ± 0.12	1 ± 0.92	1

T0:1st day T1: 3rd day T2:7th day

^*^p < 0.05

Although only six patients in the control group reported using painkillers on day one, our results indicate that there are notable variations between the groups. Our analysis revealed significant differences between all groups and time intervals, with a *p* value of less than 0.001. These findings are significant in the context of our study and emphasize the importance of carefully considering all relevant factors when analyzing data.

## Discussion

Lasers have become an indispensable tool in dentistry, serving a wide range of purposes. From surgical procedures involving the gingiva and jawbone to managing tooth decay, gingival reshaping, maxillary sinusitis, and aesthetic dental treatments, lasers have proven effective in addressing a variety of oral health issues. Additionally, they help to treat conditions such as periodontitis, sensitivity, gingival discoloration, aphthae and herpes, recurrent aphthae, jaw joint disorders, and oral mucosal diseases. Lasers also play a crucial role in sterilizing root canals, treating peri-implantitis, promoting postextraction wound healing, and facilitating implant surgeries. Owing to their precision and versatility, lasers are revolutionizing the field of dentistry and improving patient outcomes [16, 17].

Owing to their numerous advantages, soft tissue applications have seen a significant increase in the use of Nd:YAG, Er:YAG, CO_2_, and diode lasers. These lasers require minimal or no anesthesia, eliminate vibration, cause minimal scarring, and shorten the application time. Additionally, owing to their hemostatic features, they offer an expanded field of view, particularly in children. The risks of postoperative edema, pain, and infection are low, and this method does not require stitches. This makes it a highly tolerable procedure, especially in children. The use of these lasers in soft tissue applications has greatly enhanced the safety and efficacy of the procedure, making them a preferred choice among healthcare professionals [18–20].

Reports suggest that low-level laser therapy can potentially reduce inflammation and provide relief from postoperative pain. This therapy can alter the pain threshold, decrease the release of bradykinin and histamine, stimulate the production of natural endorphins, and influence prostaglandin production [21].

In a meta-analysis conducted in 2019, eight animal-based clinical studies were evaluated [10]. Out of these, four studies did not provide evidence supporting the enhancement of wound healing through Low-Level Laser Therapy (LLLT) with GaAlAs and He–Ne lasers [22–24]. Conversely, the remaining four clinical trials indicated that HF, diode, and Ga-As lasers exhibited the potential to improve wound healing under the examined conditions [14, 25–27].

According to a recent study, patients who received orthodontic arch wire placement reported less pain when undergoing low-level laser therapy (LLLT) than those who received placebo treatment. However, these results were not statistically significant (*p* > 0.05) [28].

According to the study conducted by Ismail et al. on postoperative pain after root canal treatment, the groups were divided into LLLT, laser-activated irrigation, and control groups. The control group reported the highest level of pain in 24-h pain values, followed by LAI and LLLT (*p* < 0.001). However, no significant difference was found among the three groups at 72-h measurements (*p* = 0.179). These findings suggest that while LLLT and LAI may be effective in reducing postoperative pain, further research is needed to determine their long-term efficacy and potential benefits [29].

A study conducted in 2018 found a statistically significant difference on the 7th day when the groups with and without LLLT were evaluated in 101 pediatric patients receiving chemotherapy in cases of oral mucositis. When pain perception and analgesic use were assessed on days 4 and 7, the decrease on the 7th day was found to be statistically significant (*p* < 0.007) [30]. The research results demonstrate clear parallelism with the presented findings.

According to a clinical study involving 60 pediatric patients diagnosed with minor recurrent oral aphthous stomatitis, LLLT administered for four consecutive days postdiagnosis led to a significant decrease in lesion size. Size reduction was measured using a periodontal probe, and it was observed that the lesion size decreased significantly between the fourth and seventh days (*p* < 0.05). Additionally, the study reported a statistically significant decrease in pain perception on the fourth day (*p* = 0.0001) [31].

Herascu et al. investigated the effects of LLLT on postoperative wounds. In conclusion, they reported that LLLT at a wavelength of 904 nm stimulated postoperative aseptic wound healing [32]. Recent studies have observed a notable effect of low-level laser therapy (LLLT) in vivo, specifically in increasing the phagocytic activity of macrophages during the initial stages of tissue formation following trauma. These findings have significant implications for the potential use of LLLT as a therapeutic intervention for the treatment of various injuries and for postoperative recovery [33–35].

It is crucial to emphasize the critical nature of these clinical advantages, particularly in children with weakened immune systems. This encompasses individuals with insulin-dependent diabetes who previously experienced endocarditis or heart complications, had undergone heart surgery, had artificial valves, and oncology patients undergoing chemotherapy or radiation therapy.

Following tooth extraction, rapid ridge resorption is a common occurrence that can impede the recovery of lost alveolar bone. Consequently, ridge augmentation procedures can be arduous and require the assistance of a proficient dental professional to guarantee optimal results. A recent study conducted by Akhil et al. analyzed the changes in bone density in the test (LLLT) and control groups in terms of alveolar ridge augmentation techniques. The results revealed that the test group had a change in bone density of -136 ± 236.08 HU, while the control group had a value of -44.30 ± 180.89 HU. Interestingly, there were no statistically significant differences in these parameters between the two groups [36]. Maintaining the three-dimensional dimensions of the alveolar bone is of utmost importance and can be achieved by accelerating the wound healing process. Our study confirms this perspective, as supported by our findings.

According to a study conducted by Mandic in 2015, evidence suggests that LLLT can effectively promote bone healing around immediate implants. The findings of this study indicate that LLLT may be a promising approach for improving the success rates of implant procedures. This is certainly a noteworthy development in the field of implant dentistry and could have far-reaching implications for both practitioners and patients alike [37]. Overall, it is encouraging to see that research in this area continues to advance our understanding of how we can best support optimal outcomes for those undergoing alveolar bone procedures.

In a study conducted by Abdulhameed et al., the impact of the low-intensity pulsed ultrasound method, which was indicated to possess a similar bio-stimulation mechanism to LLLT, was evaluated on marginal bone loss and osseointegration in dental implants. The research reported that, at the postoperative 6th month, the method exhibited a statistically significant inductive effect compared to the control group [38].

Furthermore, two studies demonstrated the advantageous effects of diode laser (with parameters of 20 mW and 670 nm, as well as 200 mW and 820 nm) when applied immediately following tooth extractions. This laser was found to significantly boost the development of new blood vessels, thereby facilitating wound healing [14]. Additionally, it accelerated the initial stage of wound healing by organizing the clot, which, in contrast to the control group lasting a week, was achieved within only 3 days after tooth extraction [27].

Mester and Tota [39] discovered that LLLT accelerated wound healing in rats. This is because LLLT can trigger the release of growth factors, including vascular endothelial growth factors [40]. A review of various in vivo and clinical studies, along with a meta-analysis by Woodruff et al. [41], showed that LLLT is an efficient method for enhancing wound tensile strength and reducing wound size, resulting in faster healing times. Another study by Noda et al. found that laser-treated sites had faster epithelialization than nonirradiated controls [8].

According to the study by Elbay et al., there were no statistically significant differences in pain perception between the LLLT and control groups following primary molar tooth extraction. However, the mean VAS scores were slightly higher for the control group on the first and second evenings, and the PRS scores were higher in the control group in the first evening. More analgesics were administered to children in the control group on the first evening, but both groups received equal amounts in the next two evenings [2].

In a recent study conducted by Paschoal and Santos-Pinto, parallel wound healing was observed in both the control and laser groups after premolar tooth extraction in adolescents. Although pain perception values were lower in the laser group, no statistically significant difference was observed between the two groups [9]. It is interesting to note that this clinical study produced findings that are quite similar to the results obtained in our research. Our findings are supported by those of other experts in the field.

As a limitation of our study, it should be noted that anxiety may have developed in pediatric patients before the second tooth extraction appointment following the initial extraction session.

## Conclusion

In pediatric patients, low-level laser therapy demonstrated advantages over the control group in terms of both pain perception and wound healing following tooth extraction. As the cellular mechanisms through which LLLT confers benefits become more comprehensively understood in the future, its routine dental applications are expected to expand.

## Data Availability

The datasets generated during and/or analyzed during the current study are available from the corresponding author upon reasonable request.
